# Equilibrium and kinetic modeling of Cr(VI) removal by novel tolerant bacteria species along with zero-valent iron nanoparticles

**DOI:** 10.1038/s41598-024-57835-z

**Published:** 2024-04-14

**Authors:** Shashank Garg, Simranjeet Singh, Nadeem A. Khan, Jastin Samuel, Praveen C. Ramamurthy, Joginder Singh

**Affiliations:** 1 Department of Biotechnology, Lovely Professional University, Phagwara, Punjab 144411 India; 2 Interdisciplinary Centre for Water Research (ICWaR), Indian Institute of Science, Bangalore, 560012 India; 3 Interdisciplinary Research Center for Membranes and Water Security, King Fahd University of Petroleum and Minerals, Dhahran, 31261, Saudi Arabia; 4 Waste Valorization Research Lab, Lovely Professional University, Phagwara, Punjab 144411 India; 5 Department of Botany, Nagaland University, HQRS: Lumami, Nagaland, 798627 India

**Keywords:** Hexavalent chromium, Nanobioadsorbent, Zerovalent iron, Isotherm, Modeling, Kinetics, Energy and society, Environmental economics, Environmental impact, Sustainability

## Abstract

This work describes the study of the removal of a refractory contaminant, i.e., Hexavalent chromium (Cr(VI)) from aqueous systems by a novel adsorbent comprising Cr(VI) tolerant bacteria and zero valent iron nanoparticle (nZVI). A gram-positive, rod-shaped bacteria used in the study were isolated from wastewater (WW) received from the effluent of leather industries. The adsorbents were prepared with bacteria, nZVI alone, and a combination of both. The adsorbent comprising both elements was found to remove Cr(VI) with a higher percentage (93%) and higher capacities (0.58 mg/g) as compared to adsorbent with bacteria (Cr(VI) removal = 63%, q_e_ = 0.163 mg/g) or nanoparticles (Cr(VI) removal = 80%, q_e_ = 0.45 mg/g) alone. The adsorbent worked best at neutral pH, and the removal became saturated after 90 min of incubation. Equilibrium studies with isotherm modeling suggested that the adsorption process follows sips isotherm (R^2^ = 0.9955), which is expected to bean intra-particle diffusion process before the actual adsorption. Process kinetics was modeled with pseudo-first order, pseudo-second order, and Vermeulen model. The diffusion coefficient determined by fitting the kinetic data to Vermeulen model was found to be 0.0000314 cm^2^/s. The adsorbent can be tested further for continuous flow processes to find more insights about the usage on a large scale.

## Introduction

The main reasons for increasing environmental pollution levels are the expanding population and industrialization^1,2^. Researchers are primarily concerned about water contamination resulting from industrial waste emissions containing significant amounts of organic and inorganic contaminants from industries like textile, food, dye, and paint^3–5^. Hexavalent chromium Cr(VI) is now regarded amongst the significant environmental pollutants due to its increasing use in the majority of industrial processes (leather processing, electroplating, printing, dyeing, and metallurgy), which causes disease in life forms^6–8^. Several industry unit operations produce chromium-containing chemical species, of which trivalent chromium Cr(III) and Cr(VI) are the most common. These chromium species are accumulating in natural waters due to improper disposal of these industries’ effluent water, which is deadly for plants and animals because chromium is carcinogenic and mutagenic to a certain extent^9^. This addresses the uproaring demand for sustainable, easy, and economical methods for properly disposing Cr(VI) bearing wastewater. A study on inhabitants of Kanpur, India (an area with a lot of tanneries and chromium salts manufacturing industries), has revealed that impaired hemoglobin function and gastrointestinal and dermatological symptoms are linked to elevated concentrations of Cr(VI) in groundwater^10^.

To safeguard the environment from Cr(VI) hazards, it is necessary to lessen the amount of Cr(VI) in the effluent water by treating it through certain processes before it is released into natural waters. The general physiochemical methods include adsorption^11–14^, ion exchange, and filtration^15–17^. These processes only remove the metal ions and do not convert them into a stable or non-toxic form, limiting their sustainable application^18^. The filtration process’s scalability is difficult, limiting its application in handling higher flow rates. Other methods include the application of principles of electrokinetics^19^, electrocoagulation^20^, electrochemical reduction^21^, electrodialysis^20^, and electrodeionization^22^. Cr(VI) in wastewater can also be removed by treating the water with chemicals like H_2_S^23^, sodium dithionite^24^, sodium metabisulfite^25^ sodium dichloroisocyanate and sodium hypochlorite^26^, calcium metabisulfite^27^, ferrous sulphate^28^. Biotransformation^29^, biosorption^30^, biomineralization^31^, and extracellular precipitation^32^ are a few techniques which utilizes biological organisms (plants or microbial biomass) as remedial agent for heavy metal contaminated water.

The Cr(VI) remediation processes discussed above have limitations that affect their field application potential^33^. Although simple and economical, physical techniques do not degrade or reduce Cr(VI). Similarly, chemical techniques change the oxidation state of Cr(VI). Still, in doing so, a lot of energy and consumable compounds are utilized, which adds to the generation of excessive amounts of toxic sludge, disposal of which is a question. The modern application of nanomaterial for adsorption allows high adsorption capacities, but dispersal of bare nanoparticles in environmental matrix is a limitation.

Immobilized nanomaterials answer these limitations and allow higher utilization of available area on nonmaterial surfaces^34,35^. Composites of nanomaterial like zero valent iron nanoparticle (nZVI, with high adsorption capacities and reducing capacities) with a biofilm of Cr(VI) reducing microbes (or iron-reducing bacteria) were tested in earlier reported studies yielding very high removal capacity but having the limitation of integrity and sustainability of the surface biofilm on sorbent in large scale field applications which in turn affects the technology transferability to the industry^36^. In a recent study, nZVI has been utilized to assist fenton reaction based degradation of organic dye (reactive red 198) with an efficiency of around 97%^37^. Other hybrid nanoparticles are also utilized for removal of contaminants^38^. Azari et al. have investigated removal of azithromycin with novel magnetic nanoparticles which exhibited high removal capacities^39^. Nitrobenzene was degraded with a composite of nZVI with high removal efficiencies which further strengthen the potential of nZVI in WW treatment strategies^40,41^.

In the current work, four different novel nano-bioadsorbents (NBA) have been developed by immobilizing nZVI and Cr(VI) tolerant bacteria in a combinatorial design. The different combinations of NBA were assessed for their %Cr(VI) removal capacity by varying initial pH of the solution, initial Cr(VI) concentration, adsorbent amount used in experiment, and time (one factor at a time) under synthetic wastewater. Equations and kinetic studies further evaluated the NBA with the highest %Cr(VI) capacity.

## Results and discussion

The above experiments’ results, including the isolated organism’s characteristics, effects of the operating parameters over %Cr(VI) removal, isotherm modeling, and kinetic modeling are presented below.

### Characterization of isolated Cr(VI) tolerant bacteria

**Figure 1:**
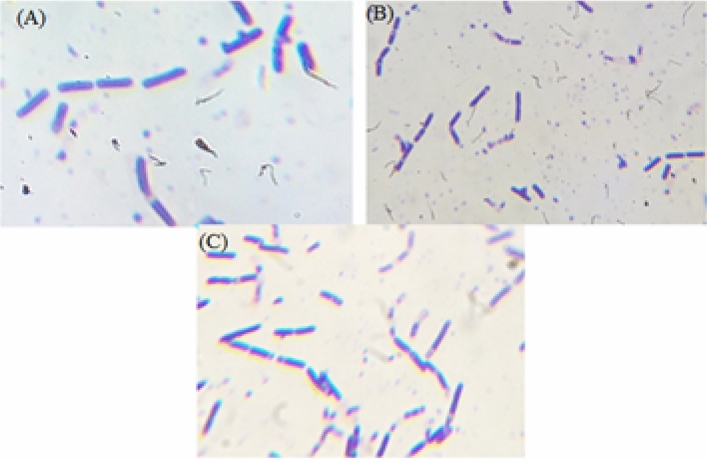
Brightfield microscopic images of isolated bacteria from tannery effluent. (Magnification × 100, all 3 are same organism).

The morphological investigation suggested that this organism is a rod-shaped bacterium. The purple-coloured colonies confirm that it is gram-positive (Fig. 1). As it is able to grow under Cr(VI) supplemented NA, it can tolerate Cr(VI). MIC is estimated to further confirm the Cr(VI) toxicity, and the results are presented below.

### Determination of MIC of Cr(VI) for the bacteria

**Figure 2:**
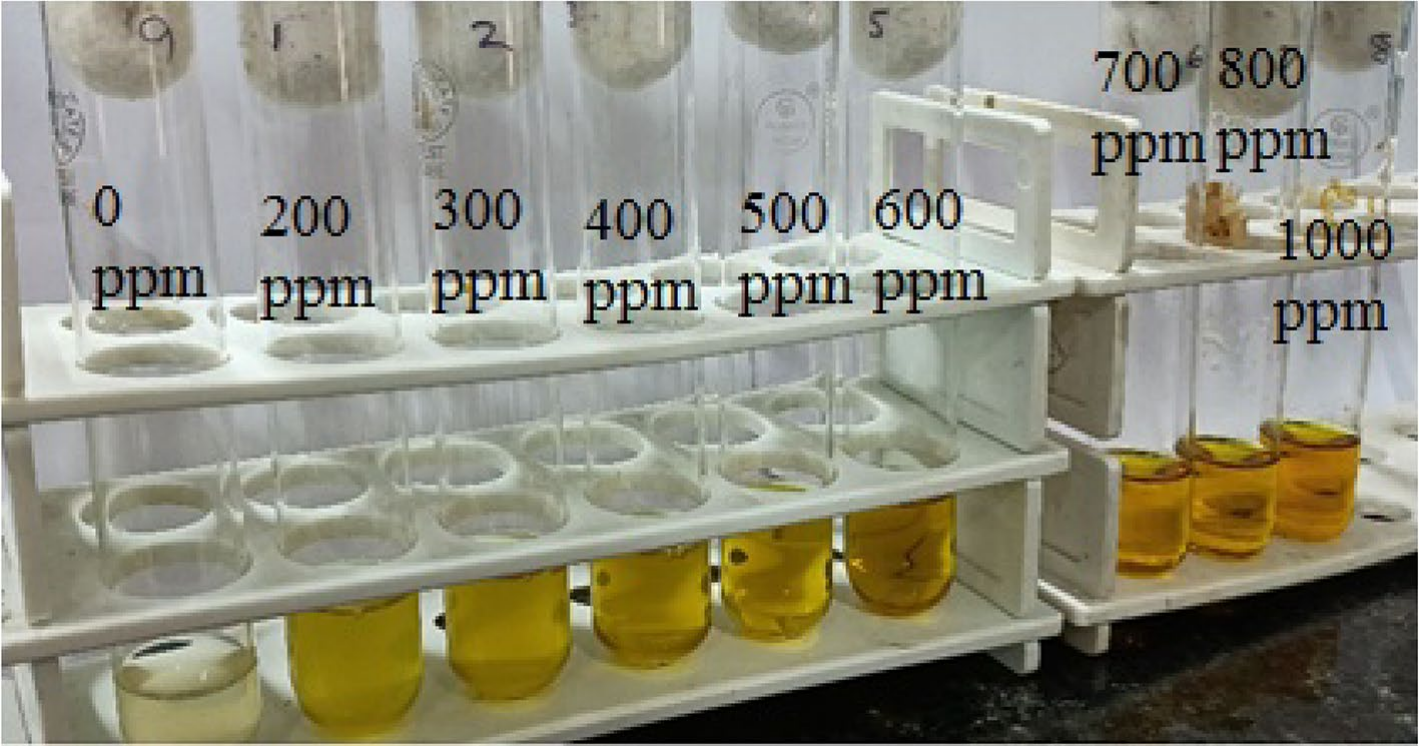
Growth of the isolate in increasing concentration of Cr(VI) supplemented nutrient broth for MIC determination. Growth can be observed upto 400 ppm.

MIC is an important property that can be preliminarily helpful in predicting the utility of the bacteria for wastewater treatment purposes. A higher MIC suggests that the organism can survive well at that concentration of the toxic molecule. In the experiment performed, the turbidity was observed in test tubes that had Cr(VI) up to 400 ppm, while the test tubes with concentrations beyond that were clear. This happened because the strain was not able to sustain beyond 400 ppm of Cr(VI) (Fig. 2). Thus the MIC of Cr(VI) for the isolate was found to be 400 ppm. Verma et al.^42^ isolated a group of microbes from tannery effluents that were able to tolerate Cr(VI) upto 200 ppm. In another study at Central Leather Research Institute (Chennai), the organisms isolated were shown to tolerate Cr(VI) upto 80 ppm^41^. The isolated organism exhibits a higher tolerance as compared to these organisms which may give a better removal percentage for Cr(VI). Gram positive rods have shown to be Cr(VI) tolerant by other studies as well. Bharagava et al. have isolated gram positive rods from tannery wastewater which were able to tolerate Cr(VI) upto 100 ppm (mg/L)^43^. In that regard our bacteria was able to tolerate Cr(VI) to a higher concentration.

### Preparation and characterization of nanobioadsorbent

**Figure 3:**
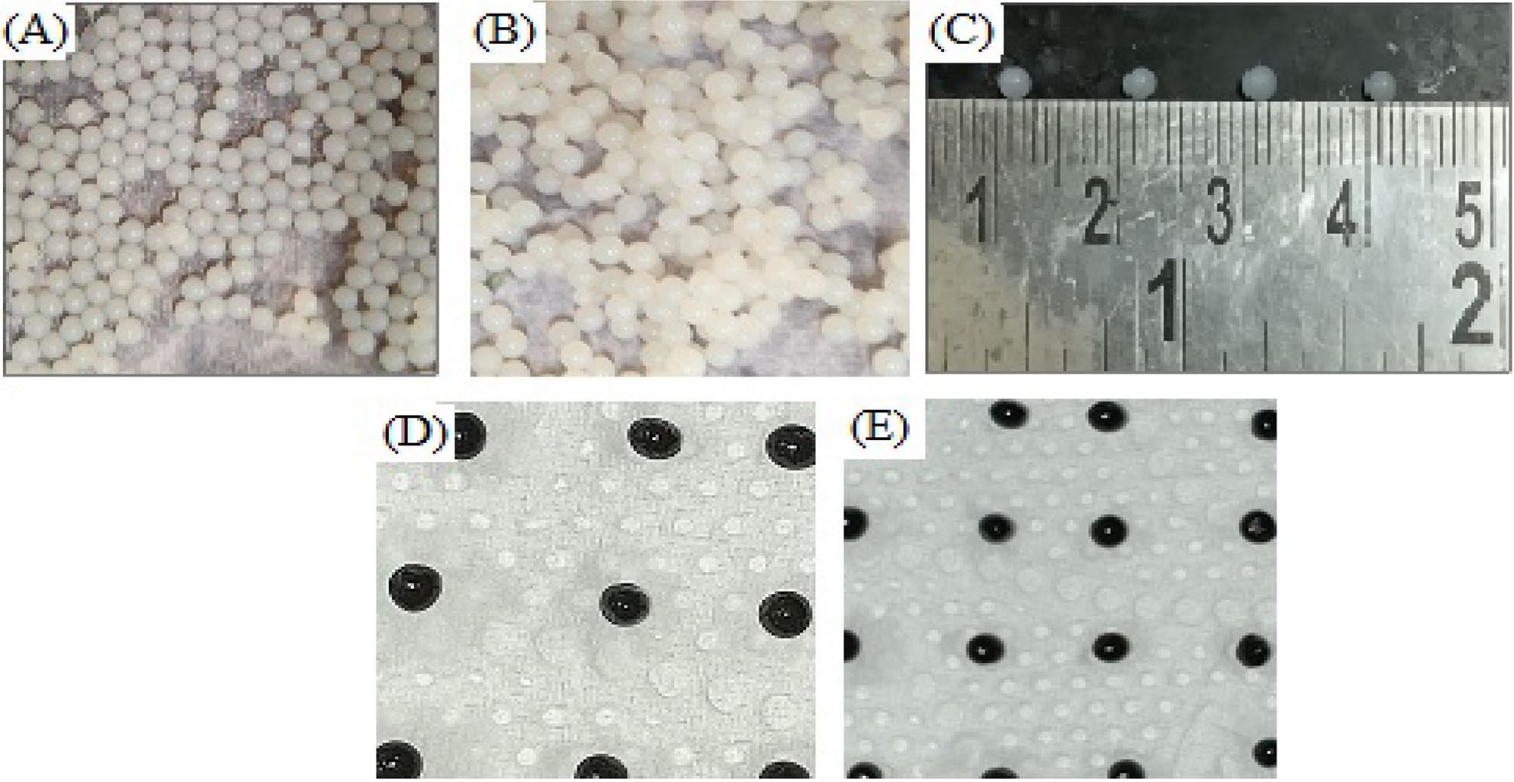
Beads of calcium alginate used as immobilization matrix (**A**) blank CA beads, (**B**) CA beads with cells, (**C**) size visualization of CA beads, (**D**) CA beads with nZVI, (**E**) CA beads with cells and nZVI.

The prepared NBAs are shown in Fig. 3. The beads displayed a homogenous, spherical geometry with an approximate diameter of 2 mm. The beads with cells and bacteria were found to be of a different color as compared to blank beads. The strength of beads was found to be enough to withstand the shear stress during the shaking of the flask.

### Effect of pH, initial Cr(VI) concentration, time of incubation, and amount of adsorbent on %Cr(VI) removal

**Figure 4:**
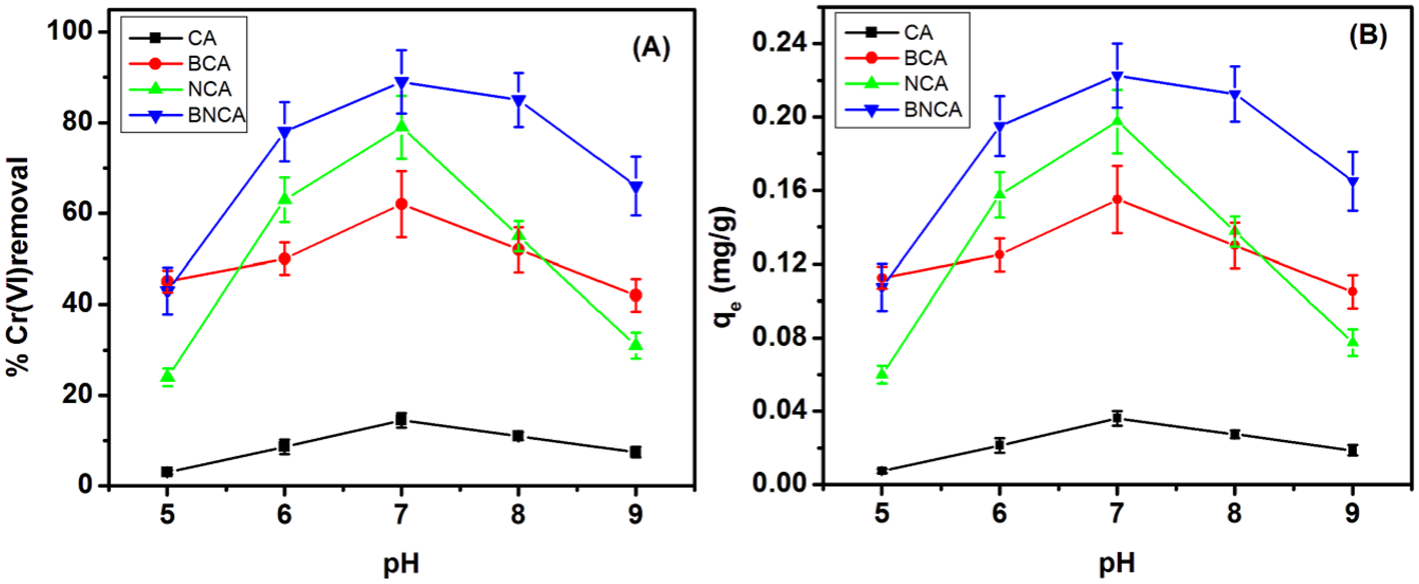
Percentage Cr(VI) removal (**A**) and q_e_ (**B**) as a function of pH for different types of adsorbents (adsorbent dosage = 2 g/L, time = 90 min, initial Cr(VI) concentration = 10 ppm).

The effect of change of pH over the % Cr(VI) removal and q_e_ is exhibited in Fig. 4A,B respectively. It can be seen that for all four types of adsorbents, the optimum pH is 7 at which the removal percentage is highest. This value of pH is used for further experiments. The removal at this pH is highest for BNCA (89%) followed by NCA (79%), BCA (62%) and is found to be lowest (14.5%) for blank beads. A some Cr(VI) was adsorbed by blank beads as well. In a similar experiment, Yu et al.^44^ reported the removal percentage is highest at a solution pH of 5. This may be attributed towards the usage of only bacterial cells for the preparation of calcium alginate beads. Li et al.^45^ observed that the stability of the nZVI in carboxy methyl cellulose-based polymer systems is highest at pH 7. This change removal percentage with pH may be due to perturbation in chemical behavior of water molecules covering the bead.

**Figure 5:**
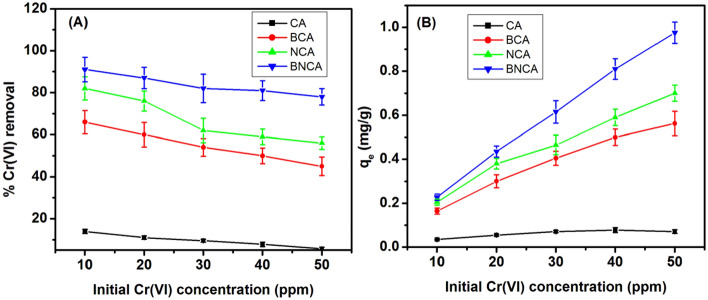
Percentage Cr(VI) removal (**A**) and q_e_ (**B**) as a function of initial Cr(VI) concentration for different types of adsorbents (adsorbent dosage = 2 g/L, time = 90 min, pH 7).

The pattern of Cr(VI) removal % and q_e_ with a change in the initial concentration of Cr(VI) is shown in Fig. 5A,B. Here, as well, the highest removal is for BNCA (91%) and the lowest is for blank beads (14%). A similar trend is observed for all types of NBA where the %Cr(VI) removal is decreasing as the initial Cr(VI) concentration increases. This is well expected because, with higher initial concentration, the residual concentration also increases as the capacity of NBA gets exhausted at lower amounts only. A similar result is obtained by other groups as well, which worked on immobilized marine yeast for uranium removal. It is also interpreted that the adsorption process is more efficient (in terms of removal percentage) for smaller concentrations of analyte i.e. Cr(VI) and nevertheless, this value is similar to that of concentration of Cr(VI) in effluent of targeted industries^46^.

**Figure 6:**
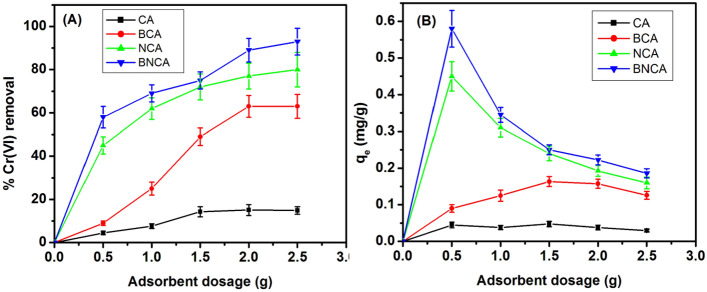
Percentage Cr(VI) removal (**A**) and q_e_ (**B**) as a function of adsorbent dosage for different types of adsorbents (pH 7, time = 90 min, initial Cr(VI) concentration = 10 ppm).

The pattern of change of residual Cr(VI)% and qe by varying adsorbent dosage is depicted in Fig. 6A,B. It is found that with an increase in the amount of adsorbent, the removal percentage is increasing significantly. Although for BNCA and NCA, a major part is adsorbed at the least amount of adsorbent (~ 50%), the increment in adsorbent dosage is affecting the removal significantly. It is also seen that for the taken Cr(VI) concentration (10 ppm), the adsorbent is getting saturated after 2 g/L of dosage, which suggests that either there is some reversible reaction which is taking place or some mass transfer limitation which is stopping the further adsorption process. It may also happen that IPD limits the diffusion from the surface into the bead in the given time (90 min). It left for more time, this movement may become possible thus increasing the removal%.

**Figure 7:**
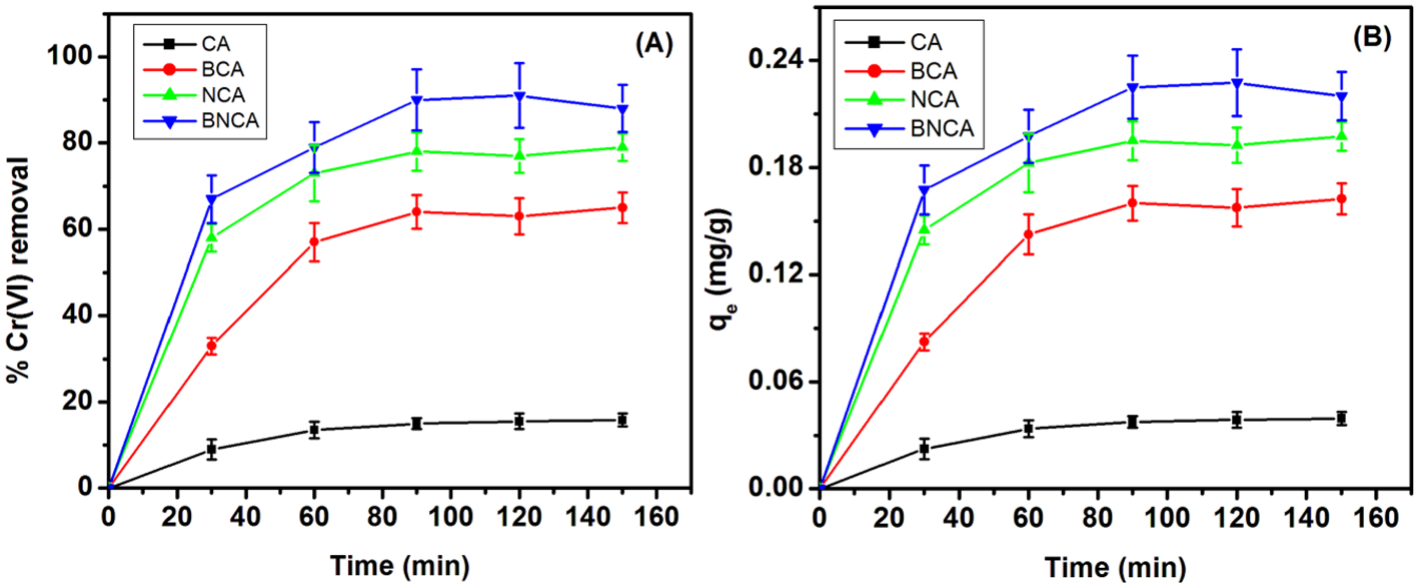
Percentage Cr(VI) removal (**A**) and q_e_ (**B**) as a with time of incubation for different types of adsorbents (adsorbent dosage = 2 g/L, pH 7, initial Cr(VI) concentration = 10 ppm).

The relationship of time with the %Cr(VI) removal and qe is depicted in Fig. 7A,B. It is observed that the adsorption process reaches a plateau after around 90 min of incubation for all the types of adsorbents and is even faster for blank beads. The analysis also suggests that the adsorbent gets saturated before 100% removal. It can also be observed that the initial rate of adsorption is faster, and there is retardation in the adsorption rate with the time, which is a feature of most similar adsorption processes^47^.

### Adsorption isotherm and modeling

**Figure 8:**
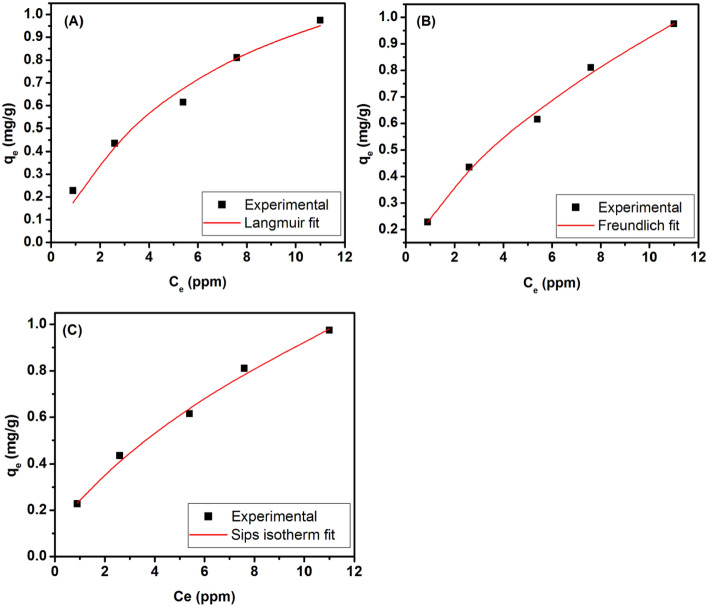
Plot of isotherm and model fitting; (**A**) non-linear fit to Langmuir isotherm. (**B**) Non-linear fit to Freundlich isotherm. (**C**) Non-linear fit to Sips isotherm.

Three different isotherm models (Langmuir, Freundlich, Sips) were utilized in this study to comprehend the nature of adsorption of Cr(VI) to BNCA. The non-linear form of experimental isotherm and its linear fitted models are depicted in Fig. 8a–c. The values of equilibrium constant and R^2^ are tabulated in Table 1. The data suggests that the isotherm is most likely to be modeled as per Sips isotherm or Frendlich isotherm. Sips isotherm is well suitable for the current adsorption systems as this model assumes that adsorption occur in a non-ideal, reversible manner that is not limited to monolayer adsorption. Sips isotherm is a sound model used to study heterogeneous systems. The operating parameters of the isotherm are a relative measure of the heterogeneity of the adsorbent^48^. Here, the slope value of around 0.2397 suggests that the surface of adsorbent is more of the homogenous kind, which is attributed towards the homogeneity of size and shape of nZVI. The comparison of values of obtained parameters is not recommended as these are highly annexed to process variables.

**Table 1:** Modeled equations of isotherms with their parameters and coefficient of determination.

Model	fitting equation	Corresponding parameters	R^2^	SSE
Langmuir	${q}_{e}=\frac{1.572\times 0.139{C}_{e}}{1+0.139{C}_{e}}$	Q_0_ = 1.572 mg/g b = 0.139 L/mg	0.9794	0.007183
Freundlich	${q}_{e}=0.242{C}_{e}^{\frac{1}{1.717}}$	K_f_ = 0.242 (mg/g) (L/g)^n^ n = 1.717	0.9954	0.001608
Sips	${q}_{e}=\frac{0.2397\times {C}_{e}^{0.5516}}{1-{0.0223C}_{e}^{0.5516}}$	K_s_ = 0.2397 L/g a_s_ = − 0.0223 L/mg β_s_ = 0.5516	0.9955	0.001578

### Kinetics of the adsorption process and its modeling

**Figure 9:**
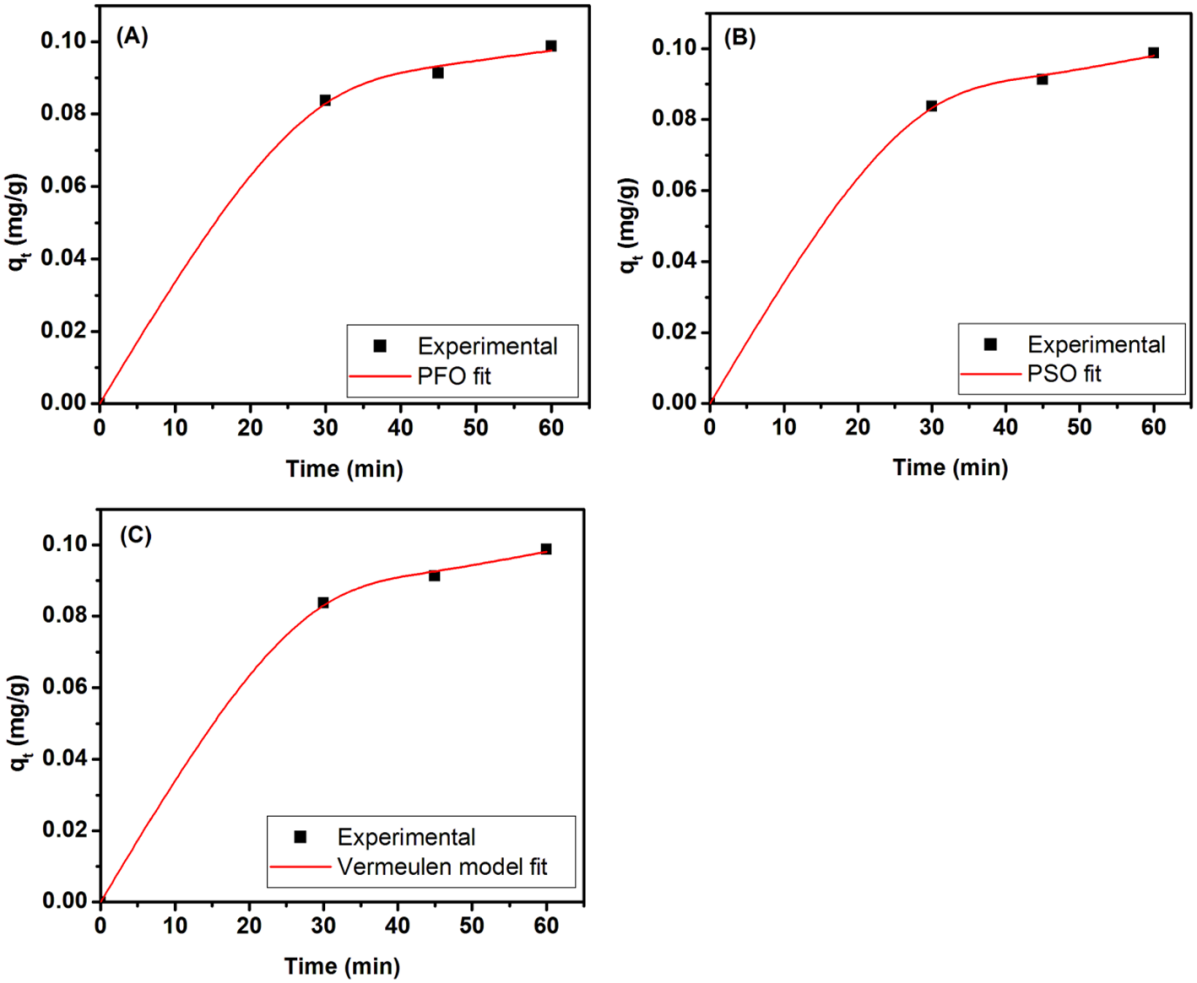
Graphs of kinetic data of the adsorption process; (**A**) non-linear fit to the PFO kinetic model, (**B**) non-linear fit to the PSO kinetic model, and (**C**) non-linear fit to the Vermeulen model.

Kinetics of adsorption is defined as time-dependent behavior of the amount of adsorption. This is a crucial parameter to analyze the behavior of the adsorption process. Kinetic parameters are useful in evaluating, designing and scaling up the systems that utilize adsorption. The intrinsic theoretical complexity of the adsorption process limits the understanding of kinetics as adsorption process is mostly an orchestra of several physiochemical processes. The model equations along with coefficients obtained after performing regression, are given in Table 2. Figure 9A depicts the linear fit of kinetic data to PFO model and Fig. 9B exhibits the fitting of the kinetic data to linear form of PSO model equation. The linear fit of the Vermeulen model is shown in Fig. 9C. Comparison of the R^2^ values of the model equations obtained after linear fitting suggests that the Vermeulen model is the better fitting model for fitting the kinetic data with a R^2^ value of 0.995 followed by PSO model (R^2^ = 0.993). The low value of the Y-intercept (0.011) in the Vermeulen model fit reveals that IPD shall be the major rate-limiting step in the entire adsorption process. Recently, Kulkarni et al.^49^ have used a modified form of Vermeulen diffusion model to study the kinetics of adsorption of Cu(II) ions onto calcium alginate beads. They argued that the mesoporous nature of their beads requires an intermediate model that will combine the features of fractal-like structure and pore diffusion effects. Yao and Chen^50^ have also suggested in their study that the Vermeulen kinetic model is better suitable to describe the cases when IPD is the rate-controlling step.

**Table 2:** Modeled equations of adsorption kinetics with its parameters and coefficient of determination.

Model	Model equation	Corresponding parameters	R^2^	SSE
PFO	${q}_{t}={q}_{e}(1-{\text{exp}}\left(-{k}_{1}t\right))$	k_1_ = 0.0578 s^−1^, q_e_ = 0.1007 mg/g	0.999053	6.02 × 10^–6^
PSO	${q}_{t}={q}_{e}\left(1-\frac{1}{1+{q}_{e}{k}_{2}t}\right)$	k_2_ = 0.6485 g/mg/s, q_e_ = 0.1191 mg/g	0.999613	2.46 × 10^–6^
Vermeulen kinetic model	$\frac{\overline{{\text{q}}}}{{{\text{q}} }_{{\text{e}}}}=\sqrt{1-{{\text{e}}}^{\left(\frac{-{\text{D}}{\uppi }^{2}{\text{t}}}{{{\text{R}}}^{2}}\right)}}$	D = 0.0000314 cm^2^/s, q_e_ = 0.1068 mg/g	0.999578	2.68 × 10^–6^

## Conclusions

The study evaluates a novel hybrid adsorbent for its Cr(VI) removal capacity. The adsorbent system was developed by immobilizing nZVI and Cr(VI) tolerant bacteria together in calcium alginate beads. The major findings of the current work are isolation of novel Cr(VI) tolerant bacteria species from WW of leather processing industry. The gram-positive rod shaped bacteria exhibited MIC of Cr(VI) for the organism was found to be 400 ppm. Analytical grade nZVI powder and bacteria were immobilized in CA beads separately and together to prepare NBA. The adsorbent was evaluated for removal of Cr(VI) from synthetic WW and was found to remove Cr(VI) with a higher percentage (93%) and higher capacities (0.58 mg/g) as compared to adsorbent with bacteria (Cr(VI) removal = 63%, q_e_ = 0.163 mg/g) or nanoparticles (Cr(VI) removal = 80%, q_e_ = 0.45 mg/g) alone. pH 7 was found to be most favourable for removing Cr(VI) from aqueous environments. Table 3 comprehends adsorption capacities of some similar studies. With time, the %Cr(VI) removal increases and gets saturated after 90 min of incubation. Isotherm modeling was performed and its was found that data fitted most appropriately to Sips model which indicates that the surface of the adsorbent was heterogeneous. The Kinetics of adsorption were best described by Vermeulen Model, and diffusion coefficient, as per theoretical calculation, was found to be 0.0000314 cm^2^/s. Further studies on fixed bed columns with synthetic WW and real water matrix are required in the same line to establish the system for industrial application and scale-up.

**Table 3:** Adorption capacities of similar adsorbents.

s no	Adsorbent	Contaminant	Maximum adsorption capacity (mg/g)	References
1	*Yarrowialipolytica* immobilized in calcium alginate	Uranium	2.25	^46^
2	Bentonite supported nZVI	Total Cr	7.3	^51^
		Pb^2+^	1.3	
		Cu^2+^	3.0	
		Zn^2+^	16.8	
3	nZVI composite	Cr(VI)	199.46	^52^
4	Polyethylene glycol-stabilized nano zero-valent iron	Cr(VI)	125.22	^53^
5	Biochar supported nZVI	Cr(VI)	24.08	^54^
6	Immobilized nZVI with bacteria	Cr(VI)	0.58	This study

## Materials and methods

### Isolation and characterization of Cr(VI) tolerant bacteria from tannery effluent

Tannery wastewater (WW) was collected from an incoming stream to the Common Effluent Treatment Plant located at Leather Complex in Jalandhar, Punjab. WW was kept in pre-sterilized amber-colored glass bottle, brought to laboratory, and processed on same day. Nutrient agar plates with different concentrations of Cr(VI) (400 ppm, 600 ppm, 800 ppm) were prepared and the WW samples (diluted with distilled water) were spread on it. Incubation of plates was done for 24 h at 37 °C. Distinct colonies were observed, picked up, and subsequently subcultured on NA supplemented with 400 ppm Cr(VI). A single colony from this plate was picked up (SG1) and used in experiments.

### Analytical method to determine minimum inhibitory concentration (MIC) of Cr(VI)

To determine MIC of Cr(VI) for the selected bacteria, 10 mL nutrient broth (NB) that was supplemented with varying amounts of Cr(VI) (200–1000 ppm), were inoculated with 100 μL of bacterial culture grown up to 0.1 OD at 600 nm. The tubes were then incubated at 37 °C in a shaker incubator and observed for growth up to 48 h. Theminimum concentration of Cr(VI) in the tube, which reflects no turbidity, is regarded as MIC for the organism.

### Analytical method to estimate Cr(VI) in solutions

To estimate Cr(VI) concentration in the working experiments, standard method of reaction with diphenyl carbazide (DPC) was used^36^. Firstly, standard reagent of DPC was prepared by dissolving 250 mg of DPC in 100 mL of pure acetone. Standards of chromium containing 0.5, 1.0, 1.5, 2.0, and 2.5 ppm of Cr(VI) were prepared by diluting 10 ppm of stock accordingly. Distilled water was taken as blank. 1 mL of each of these standards was mixed with 330 μL of 6 M H_2_SO_4_ and mixed well with a vortex mixer. To this 400 μL of DPC reagent was added to the mixed well. The optical density of pink-colored solutions thus obtained is measured at 540 nm against water treated with DPC as blank. The data obtained is used to plot a standard curve, which was further used to interpolate the concentrations of unknown test solutions. The test solutions were diluted when and as required to get the optical densities between 0.2 and 0.9 and the concentration deduced is then multiplied with appropriate dilution factor.

### Preparation of nanobioadsorbent (NBA)

nZVI was purchased from Sigma Aldrich (Ajanta Scientific Works, Amritsar, Punjab, product no 746851). It was black in color, amorphous, and hygroscopic. Calcium alginate (CA) beads were used as immobilization matrix for encapsulating bacteria and nZVI to prepare the NBA. Four combinations were done to prepare beads for the experiments. First, blank beads (CA) with no bacteria and no NP immobilized inside the bead. Second, beads with 0.5% (w/w) bacteria are designated as BCA. Then, beads with 0.1% nZVI (w/w) are called NCA, and beads with 0.5% bacteria + 0.1% nZVI (w/w) are named BNCA. For the preparation of beads, 2% sodium alginate (Hi Media) solution was prepared with a respective concentration of different components and beads of CA were prepared by dropping this solution in 0.2 M Calcium Chloride solution with 20-gauge needle. Beads were formed instantaneously and cured for 4 h in CaCl_2_ before being used for adsorption.

### Effect of initial solution pH, NBA dosage, time, and initial Cr(VI) concentration chromium removal by the prepared NBA

In batch studies, the effects of starting solution pH, NBA dose, duration, and initial Cr(VI) concentration on the percentage of Cr(VI) removed and the adsorption capacity were observed using all four forms of NBA (CA, BCA, NCA, and BNCA). Batch experiments were conducted in a shake flask with 50 mL volume with one variable at one time approach. pH range was taken from 3 to 8. NBA dosage was taken as 0.5, 1.0, 1.5, 2.0, 2.5 g. Time was varied from 30 to 150 min with 30 min gap in between, and beginning concentration of Cr(VI) was varied as 10 ppm to 50 ppm with an increment of 10 ppm. While varying one factor, the other factors were kept constant (pH 7, adsorbant dosage = 2 g**/**L, Time = 90 min, initial Cr(VI) = 10 ppm). %Cr(VI) removal (Eq. 1) and adsorption capacity (Eq. 2) are calculated as follows:

1 $$\% Cr\left(VI\right) Removal=\frac{({C}_{0}-{C}_{t})\times 100}{{C}_{0}},$$

2 $$q=\frac{{(C}_{0}-{C}_{t})\times V}{m},$$

where C_0_ = Initial Cr(VI) concentration in solution, C_t_ = Cr(VI) concentration in solution at any time “t”, V = Volume of the test solution, m = mass of adsorbent in the test solution.

The type of adsorbent, which was found to have the highest adsorption capacity and highest % removal of Cr(VI), was chosen for further analysis.

### Plotting of adsorption isotherm and model analysis

Adsorption isotherm occupies an important space in adsorption studies^55,56^. They are useful in providing several insights into the adsorption mechanisms and mode of adsorption^57,58^. In this study, we plotted adsorption isotherms and then tried to fit these isotherms into three different isotherm models, viz. Langmuir (Eq. 3), Freundlich (Eq. 4), and Sips (Eq. 5). The model equations are given below.

3 $${q}_{e}=\frac{{Q}_{0}b{C}_{e}}{1+b{C}_{e}},$$

4 $${q}_{e}={K}_{F}{C}_{e}^\frac{1}{n},$$

5 $${q}_{e}=\frac{{K}_{s}{C}_{e}^{{\beta }_{s}}}{1+{a}_{s}{C}_{e}^{{\beta }_{s}}}.$$

Here, “Q_0_” is maximum monolayer coverage capacities (mg/g), “b” is Langmuir isotherm constant (mL/mg), “K_F_” is Freundlich isotherm constant (mg/g) (mL/g)^n^ related to adsorption capacity, “n” is adsorption intensity, “K_s_” is Sips isotherm model constant (L/g), “β_s_” is Sips isotherm model exponent, “a_s_” is Sips isotherm model constant (L/mg)^59^.

Non-linear regression were performed by minimising the Sum of Squares of Errors (SSE) to deduce the relevant isotherm model parameters and goodness of the fit was estimated by calculating the value of the coefficient of determination (R^2^)^60^. To plot an isotherm, 50 mL, 20 ppm Cr(VI) solution was incubated with varying amounts of NBA (BNCA) at pH 7. The analyte concentration was determined after 180 min of incubation and residual concentration at equilibrium (C_e_), and adsorption capacity at equilibrium (q_e_) (Eq. 6) was calculated as follows.

6 $${q}_{e} =\frac{{(C}_{0}-{C}_{e})\times V}{m}.$$

### Kinetic analysis of the adsorption process

Kinetic analysis refers to the study of change in the removal percentage of the adsorbate (Cr(VI)) with time. To perform kinetic analysis 2 g of adsorbent (BNCA) was inoculated in 50 mL of 10 ppm Cr(VI) solution and stirred at 100 rpm. The initial pH of solution was set to 7. Regular sampling was done at given time points and Cr(VI) concentration was determined using standard method of DPC, as stated above. The data obtained was used to calculate Cr(VI) removal percentage.

The data obtained from the experiment was further utilized to perform a kinetic modeling of the process. We have used three different model viz. Pseudo First order (PFO) (Eq. 7), Pseudo second order (PSO) (Eq. 8), and Vermeulen Model (Eqs. 9, 10) to model the given data. PFO and PSO are commonest models used in modeling of kinetic data in adsorption studies^61^. Along with these two, the reason behind choosing Vermeulen Model is that it assumes interparticle diffusion (IPD) phenomenon, which is well suitable for the case of diffusion inside the beads. As we have a CA bead, a macro particle with pores inside it, we find this a suitable modelfor this study involving kinetic study. The equations of the models are as follow:

7 $${q}_{t}={q}_{e}\left(1-{\text{exp}}\left(-{k}_{1}t\right)\right),$$

8 $${q}_{t}={q}_{e}\left(1-\frac{1}{1+{q}_{e}{k}_{2}t}\right),$$

9 $$\frac{\overline{{\text{q}}}}{{{\text{q}} }_{{\text{e}}}}=\sqrt{1-{{\text{e}}}^{\left(\frac{-{\text{D}}{\uppi }^{2}{\text{t}}}{{{\text{R}}}^{2}}\right)}}.$$

The linear form of the equation is

10 $${\text{ln}}\left(1-{\left(\frac{\overline{q}}{{q }_{e}}\right)}^{2}\right)=\frac{-{\text{D}}{\uppi }^{2}{\text{t}}}{{{\text{R}}}^{2}}.$$

Here, “D” is diffusion coefficient, $\overline{{\text{q}} }$ is the average value of q in the spherical particle of radius R at any particular time.

### Statistical analysis

Statistical analysis was performed with GraphPad PRISM (v 8.0) and MS Excel (v 2018). Non linear regression were performed with MS Excel (v 2018). For performing regression, minimization of the sum of squares of errors (SSE) was used as the standard algorithm. Final equations were represented and analyzed to get the coefficients of equations. R^2^ was used to evaluate the fitting standard of the model.

## Data Availability

The data that support the findings of this study are available from [Simranjeet Singh]. Still, restrictions apply to the availability of these data, which were used under license for the current study, and so are not publicly available. However, data are available from the authors upon reasonable request and with permission of [Simranjeet Singh].
